# Ancient duplications and grass-specific transposition influenced the evolution of LEAFY transcription factor genes

**DOI:** 10.1038/s42003-019-0469-4

**Published:** 2019-06-21

**Authors:** Bei Gao, Moxian Chen, Xiaoshuang Li, Jianhua Zhang

**Affiliations:** 1 School of Life Sciences, The Chinese University of Hong Kong, Hong Kong, China; 2 State Key Laboratory of Agrobiotechnology, The Chinese University of Hong Kong, Hong Kong, China; 3 Shenzhen Research Institute, The Chinese University of Hong Kong, Shenzhen, China; 4 Key Laboratory of Biogeography and Bioresource, Xinjiang Institute of Ecology and Geography, Chinese Academy of Sciences, Urumqi, 830011 China; 5 Department of Biology, Faculty of Science, Hong Kong Baptist University, Hong Kong, China

**Keywords:** Molecular evolution, Phylogenetics, Comparative genomics

## Abstract

The LFY transcription factor gene family are important in the promotion of cell proliferation and floral development. Understanding their evolution offers an insight into floral development in plant evolution. Though a promiscuous transition intermediate and a gene duplication event within the *LFY* family had been identified previously, the early evolutionary path of this family remained elusive. Here, we reconstructed the *LFY* family phylogeny using maximum-likelihood and Bayesian inference methods incorporating *LFY* genes from all major lineages of streptophytes. The well-resolved phylogeny unveiled a high-confidence duplication event before the functional divergence of types I and II *LFY* genes in the ancestry of liverworts, mosses and tracheophytes, supporting sub-functionalization of an ancestral promiscuous gene. The identification of promiscuous genes in *Osmunda* suggested promiscuous *LFY* genes experienced an ancient transient duplication. Genomic synteny comparisons demonstrated a deep genomic positional conservation of *LFY* genes and an ancestral lineage-specific transposition activity in grasses.

## Abstract (web summary)

Bei Gao et al. reconstruct the LEAFY gene family phylogeny in streptophytes to probe the timing of an ancestral gene duplication event. They find deeply conserved genomic syntenies of the LEAFY genes, with a lineage-specific transposition in grasses.

## Introduction

The *LFY* gene in *Arabidopsis thaliana* and its homologs constitute a plant-specific transcription factor gene family. Early functional studies in both *FLORICAULA*/*FLO* of *Antirrhinum majus*^1,2^ and *LEAFY*/*LFY* from *A. thaliana*^3^ revealed that LFY provide a key switch from vegetative to reproductive development by regulating flower development. *FLO* is expressed at the very early stages of flower development and a mutation in the gene sequence results in the failure of the transition from inflorescence to floral meristems^1,2^. Similar phenotypes were observed in the *A. thaliana lfy* mutant^3^. The expression of *LFY* in single-cell layers was able to exert long-range stimuli to activate downstream homeotic genes in all layers of floral meristems^4^, accompanied by movement of the protein into adjacent cells^5^. Besides *APETALA1* (*AP1*) and its close homolog *CAULIFLOWER* (*CAL*), genes regulated by *LFY* also include MYB and bZIP transcription factors^6^. Upon flowering, *LFY* activates *AP1*, which activates *SEP3* that together with *LFY* activates *AG*, *AP3*, and *PI* genes^7^. In this way, *LFY* and MADS-box genes constitute the feed-forward loop controlling the flowering process^8^.

However, distinct functions of the LFY homolog in rice (delineated as *RFL* or *APO2/ABERRANT PANICLE ORGANIZATION* 2) were reported^9–11^. The function of *RFL/APO2* was determined to be primarily involved in panicle branching^9^, and functioned upstream of *OsSOC1*^11^. In contrast, *LFY* acts downstream of *SOC1* to promote flowering^12^. The expression of RFL was not able to complement the phenotype of *A. thaliana lfy* mutants^9,13^. Whereas some conserved regulatory mechanisms still exist between *LFY* and *RFL/APO2*, for example, *LFY* interacts with its co-regulator *UFO* (*UNUSUAL FLORAL ORGANS*) in *Arabidopsis*^14^, while the rice RFL/APO2 can interact with *APO1*^10^, an ortholog of *UFO*. The interaction between *LFY* and *UFO* homologs were reported in several other eudicots, such as petunia^15^, *A. majus*^16^ and pea^17,18^. These studies revealed both partial functional divergence and conservation of *LFY* between rice and *Arabidopsis*.

The presence of two paralogs, *LFY* and *NEEDLY* clades, in parallel with spermatophyte polyploidy events^19^, seems to be common in gymnosperms^20^. In *Pinus radiata*, two LFY homologs were identified, with *PRFLL* predominantly expressed in male cones and *NLY* in female cones: both are expressed in vegetative meristems^21,22^. Vegetative expression of *LFY* genes has also been reported in several angiosperms^17,23,24^. Two *LFY* homologs from the lycophyte *Isoetes* L. (*IsLFY1* and *IsLFY2*) were observed to accumulate in both reproductive and vegetative tissues and are highly expressed in juvenile tissues^25^. Two *LFY* genes from the moss *Physcomitrella patens* were demonstrated to regulate the first mitotic cell division in zygotes^26^, while the *LFY* genes from the fern *Ceratopteris richardii* are required to maintain apical stem cell activity during shoot development^27^. These observations across distinct plant lineages, suggest that the non-reproductive functions of *LFY* may be ancestral^28^ and make it possible to infer an evolutionary trajectory for this family.

Combined, LFY and its homologs represented an important gene family that promotes cell proliferation and which appears to have been progressively co-opted during evolution, adapted and specialized as more complex plant structures emerged^27^. For simplicity, in this broad-scale phylogenomic study, we refer to *LFY* and its homologs as *LEAFY/LFY* genes throughout, to avoid obfuscation generated by the presence of several other gene names reported in the literature.

DNA-binding site identification and characterization accompanied by the crystal structure resolution of *LFY* identified a promiscuous intermediate form in hornworts which exhibited multiple (type I, II, and III) DNA motif-binding specificities^29^. This observation was suggested to represent a smoothing of the evolutionary transitions of *LFY* to develop new binding specificities while remaining as a single-copy gene^29^. However, Brunkard et al. commented on the promiscuous transition and provided evidence of a moss (*Polytrichum commune*) with both type I and type II *LFY* homologs and proposed that gene duplications occurred in *LFY*’s evolutionary past^30^. Brockington et al. contested the gene duplication argument by providing extra phylogenetic and ancestral state reconstruction data, and suggested there was no solid evidence to disprove *LFY* had evolved through the promiscuous transition intermediate but did not deny that gene duplications may have occurred in the past^31^. Thus the early evolutionary history of *LFY* remains an open question, especially as the existence of ancestral duplications in the evolutionary past of the *LFY* family remains to be determined.

Unlike the MADS-box and Vascular One-Zinc finger (VOZ) transcription factors, two gene families that also control flowering, that have undergone duplications following paleo-polyploidy events^32,33^, *LFY* genes have largely restored to a single copy in angiosperms after recurrent rounds of paleo-polyploidy events^34^, and have represented a valuable molecular marker for species phylogeny reconstructions^35^. Augmented transcriptomic and genomic data in diverse plant lineages suggested both the MIKC-type MADS-box genes, and the *LFY* genes have an origin in charophytes (streptophytic algae)^36^, a paraphyletic clade that represents successive sister lineages to land plants (i.e., embryophytes)^37,38^.

In this study, we mined the 1KP and Phytozome databases to identify *LFY* transcription factors. High-quality gene family phylogenies were reconstructed, including *LFY* members from seed plants, ferns, lycophytes, mosses, liverworts, hornworts, and charophytes (all in Streptophyta). The identification of both type I and type II *LFY* members from early-diverging moss lineages and liverworts, together with careful sequence alignments and a newly resolved gene family phylogeny supported an ancestral duplication of *LFY* gene family in the ancestry of mosses, liverworts, and tracheophytes. The two promiscuous *LFY* genes identified in *Osmunda* (an early diverging fern genus) were placed outside of the type I plus type II clade and clustered with hornwort genes, suggesting the promiscuous *LFY* genes might have experienced an ancient transient gene duplication. Our *LFY* family phylogeny together with the ancestral state reconstruction results support the evolutionary regime of sub-functionalization following gene duplication when the gene trees were reconciled to the hornwort-sister land plant phylogeny. By genomic synteny alignments and genomic synteny network construction, *LFY* genes were demonstrated to reside and maintained in conserved genomic regions across different angiosperm lineages, an isolated synteny network among grasses, however, was conspicuous and appears to reveal an ancestral transposition of *LFY* genes in the ancestor of grasses.

## Results

### General characteristics of *LFY* and family phylogeny

**Fig. 1:**
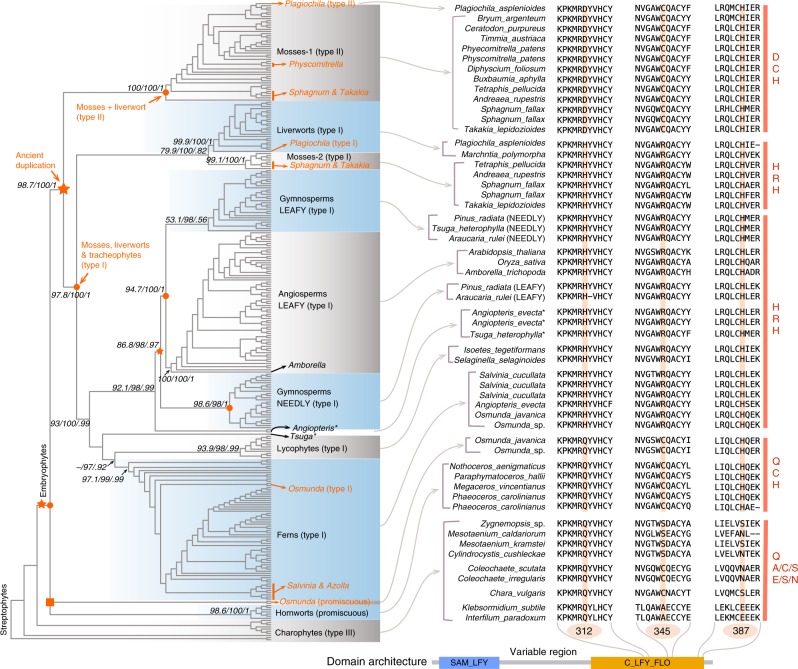
Phylogeny and diversity of LFY transcription factors in plants. The left panel depicts the LFY gene family phylogeny reconstructed under the Jones–Taylor–Thornton (JTT) + G + I substitutional model using IQ-TREE (Supplementary Data 4) and MrBayes (Supplementary Data 5). The supporting values were shown for branches in the following order: SH-aLRT test/bootstrap value/Bayesian posterior probabilities, “−“ denotes supporting values lower than 50%. The placement of two promiscuous LFY in *Osmunda* were presented as unresolved polytomy (labeled as orange square) because maximum-likelihood (sister to type I plus type II clade) and Bayesian inference (sister to hornwort promiscuous genes) methods generated different tree topologies. The right panel shows the sequence alignment results (Supplementary Data 3), focusing on the three critical amino acid sites (312, 345, and 387) which defined different types of LFY genes, and domain architecture of LFY were depicted at the bottom

In total, we collected 298 *LFY* transcription factors (Supplementary Data 1 and Supplementary Data 2) with intact *LFY* domains (NCBI-CDD database) covering all major plant lineages from charophytes to angiosperms (Fig. 1). Protein sequences of LFY transcription factors are highly conserved across multiple plant lineages (Fig. 1; Supplementary Data 3) and are characterized by two Pfam domain profiles: the N-terminal SAM_FLY domain^39^ and the C_LFY_FLO DNA-binding domain at the C-terminal region, and a variable region between the two signature domains (Fig. 1). Protein motif analyses generated consistent sequence signatures, where motifs 1, 2, and 3 constituted the SAM_FLY domain and motifs 5 through 9 are congruent to the C_LFY_FLO domain (Supplementary Figs 1–9). Interestingly, a stretch of amino acid sequence to the N-terminal of the C_LFY_FLO domain demonstrated to be less conserved in charophytes and most angiosperms, but highly conserved (motif-4 in Supplementary Figs 1–9) in all non-flowering embryophytes. The motif-4 was only observed in a single *LFY* sequence from duckweed (*Spirodela polyrhiza*, Alismatales), an aquatic plant. Analysis of the expression profiles of *LFY* in different tissues of *A. thaliana* and *O. sativa*, *LFY* indicated predominant expression in floral meristems and panicles (Supplementary Figs 10–13), consistent with previous studies demonstrating their roles in floral development in *Arabidopsis*^3^ and panicle branching in rice^9^, respectively.

Homologs of *LFY* transcription factors were absent in many genomes of chlorophytes but could be detected in *Mesostigma viride* (Mesostigmatales, early diverging charophytes), suggesting its early emergence in streptophytes (including charophytes and land plants)^36^. Those *LFY* homologs that could be identified in charophytes were incorporated in our phylogenetic analyses, including sequences from Klebsormidiales, Charales, Coleochaetales, and Zygnematales. Overall, the maximum-likelihood (Supplementary Data 4) and Bayesian (Supplementary Data 5) *LFY* gene family trees were largely consistent to the established plant phylogeny^40^. *LFY* genes from charophytes and hornworts were placed at the root position of the gene tree, followed by mosses and liverworts that constituted a direct sister clade to all type I genes from tracheophytes. Two monophyletic clades of ferns and lycophytes were well-supported as closest sisters to the genes from seed plants. In most cases, two paralogues could be detected in each of the gymnosperms and were separated into two subfamilies delineated as *LFY* and *NEEDLY*, consistent with the ancestral seed plant duplication event^19,20^. All *LFY* genes from angiosperms were clustered into one monophyletic clade, with the *Amborella LFY* gene located at the basal-most position (Fig. 1). In most angiosperms, *LFY* genes were observed to have restored to a single copy after recurrent paleo-polyploidy duplication events^34^, suggesting that the *LFY* gene might be dosage-balance sensitive^41^. However, multiple copies of *LFY* could be observed in some recent polyploids (e.g., *Zea mays, Glycine max*). Two *LFY* family members were identified in the three *Cucurbita* species (*C. maxima*, *C. moschata*, and *C. pepo*), and the local tree topology (Supplementary Fig. 14) supported a re-recognized cucurbits genome duplication event^42^.

The position of some fern and conifer *LFY* genes in the generated phylogeny was not consistent with the phylogeny of major land plant clades. Two promiscuous *LFY* genes from *Osmunda* (*Osmunda javanica* and *Osmund* sp.^43^) were placed sister to mosses, liverworts, and tracheophytes (type I plus type II) in the maximum-likelihood tree and sister to the hornwort promiscuous genes in Bayesian inference analyses (Supplementary Fig. 15 and polytomy node in Fig. 1), and the other two type I homologs from *Osmunda* were placed within the fern clade. One of two *LFY* genes from *Tsuga heterophylla* (conifer) was recognized as sister to lycophytes and ferns, while the other copy resolved in the gymnosperm (*NEEDLY*) clade. Three *LFY* homologs were found in *Angiopteris evecta* (fern), and two were placed as direct sister to seed plants and the other in the fern clade. All of genes from *A. evecta* and *T. heterophylla* are clustered within the tracheophyte clade, and all type I *LFY* members constitute a high-confidence monophyletic clade (Fig. 1).

### Ancient gene duplications of *LFY*

Sayou et al.^29^ established that the binding motif specificity and classification of *LFY* transcription factors are determined by three critical amino acid sites (312, 345, and 387) in the DNA-binding domain. In accordance with this classification, we generated detailed sequence alignments focusing on the three sites and aligned them to the family phylogeny (Fig. 1). The sequence alignments are consistent with previous studies^29,30^, the three amino acid sites are more diverse in charophytes but are more conserved in embryophytes and were fixed in distinct embryophyte lineages.

**Fig. 2:**
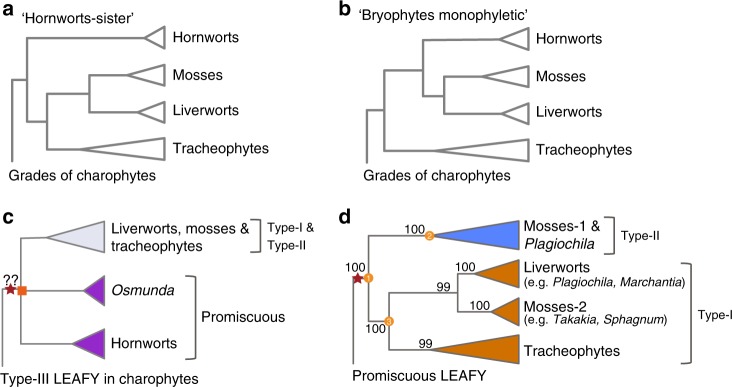
Ancient gene duplications in *LFY* inferred from gene-tree species-tree reconciliations. **a**, **b** The two currently widely accepted alternative species phylogenies of “bryophytes” according to recent phylogenomic studies. The LFY gene family phylogeny was largely in parallel with the “hornworts-sister” phylogeny. **c** A possible transient gene duplication (red star) before the functional diversification of promiscuous LFY and type I/II LFY was supported by paralogues from *Osmunda* (both type I and promiscuous). The polytomy node representing uncertainty in the phylogenetic position of *Osmunda* promiscuous LFY genes was indicated as orange square. **d** Another gene duplication (red star) shared by mosses, liverworts, and tracheophytes were well supported by paralogues from mosses and liverwort, consistent to the diversification of type I and type II LFY genes

We detected two *LFY* homologs in *Osmunda* which were categorized as promiscuous genes. Although the phylogenetic placement of the two promiscuous *LFY* genes from *Osmunda* remained unresolved (depicted as polytomy node in Fig. 1), they clustered outside of the clade containing all type I and type II genes in both maximum-likelihood and Bayesian analyses, while the two type I members from *Osmunda* were identified and placed within the fern clade (Fig. 1). While the promiscuous *LFY* genes present in hornworts have smoothed the transition of plants from water to land^29^, this observation suggest that the promiscuous *LFY* has likely experienced an ancient transient duplication and that duplicated paralogues were promptly lost in the ancestor for most land plant lineages. The paralogues were retained and resurfaced in an early diverging fern genus, *Osmunda*. We also noticed alternative land plant phylogenies which are still in play and currently interpreted as either hornworts-sister (Fig. 2a) or bryophytes-monophyletic (Fig. 2b)^40,44^, but in both cases the suggested ancient transient duplication in the ancestor of embryophytes supported by paralogues from *Osmunda* was unaffected (Fig. 2c).

Within mosses, the class Bryopsida (including the moss model *Phycomitrella patens*) is postulated to be the most specious lineage^45^. In our phylogenetic analyses, *LFY* members from the class Bryopsida were identified as type II genes and placed within the Mosses-1 clade (Fig. 1). However, in mosses outside of the Bryopsida, both type I and type II members were widely located, such as *Tetraphis* (class Tetraphidopsida), *Andreaea* (class Andreaeopsida), *Sphagnum* (class Sphagnopsida), and *Takakia* (class Takakiopsida), all of which contained both type I and type II gene members. Notably, all the non-Bryopsida moss lineages containing both type I and type II *LFY* genes were phylogenetically classified as early diverging mosses that branched away from the Bryopsida very early in the evolution of this group^45^. The four intact *LFY* genes (two type I and two type II) identified from the *Sphagnum fallax* genome were consistent with the previously reported Sphagnopsida genome duplication event (Supplementary Fig. 14)^46^.

In our reconstructed family phylogeny, the type I *LFY* members from mosses constituted the Moss-2 clade and clustered with all other type I members from liverworts, lycophytes, ferns, and seed plants (Fig. 1), constituting the large monophyletic type I clade with 100% bootstrap support value and posterior possibility in maximum-likelihood and Bayesian analyses, respectively. Though type II *LFY* members were primarily found in mosses (Mosses-1 clade in Fig. 1), we also identified one type II member from the liverwort *Plagiochila asplenioides* that clustered with type II members from mosses. All the type II *LFY* members from mosses and liverwort constituted a monophyletic clade with 100% nodal support (Fig. 1). Intriguingly, a type I paralogue found in *Plagiochila asplenioides* placed within the liverworts (type I) lineage.

Based on the reconstructed tree topology, the type I and type II clades constituted two child clades congruent with an ancient gene duplication event, which was well-supported by duplicated paralogues from mosses and the liverwort (Figs 1, 2d). Genes analyzed from hornworts are all promiscuous and placed outside of the type I and type II clades, so the ancient gene duplication occurred probably in the ancestry of mosses, liverworts, and tracheophytes and was not an event shared with hornworts; which is well-supported by the *LFY* family phylogeny when it is reconciled to the hornworts-sister land plant phylogeny (Fig. 2a).

**Fig. 3:**
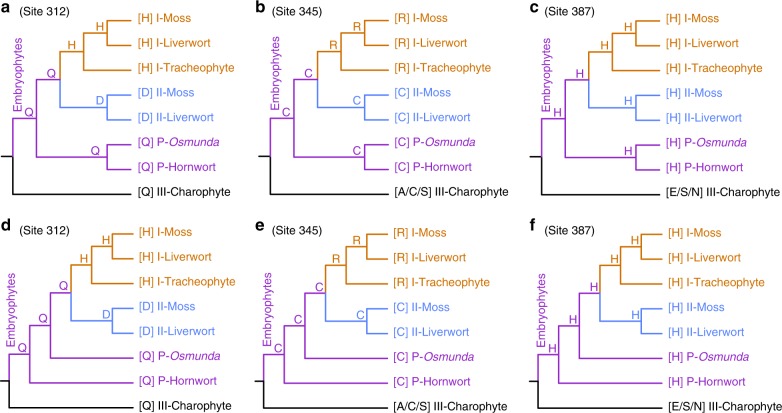
Ancestral state inference of key residues in LFY genes based on the newly resolved family phylogeny. The ancestral state of the three key amino acids were inferred and labeled on the corresponding nodes. Type I, type II, and promiscuous type *LFY* genes were depicted as yellow, blue, and purple branches, respectively. Two different simplified tree topologies were assumed considering the phylogenetic uncertainty of promiscuous type genes from *Osmunda*, where they were placed as sister to type I plus type II genes in maximum-likelihood analyses (**a**–**c**) or sister to the hornwort promiscuous genes in Bayesian analyses (**d**–**f**)

Attempts to reconstruct the ancestral state of the LFY gene family using the gene family phylogeny focusing the three amino acid sites had been reported previously^29–31^. We conducted ancestral state reconstruction analyses using the maximum-likelihood algorithm described herein, based on the same three amino acid sites and with the two alternative gene tree topologies generated in this study (Fig. 3). The ancestor of the type I and type II *LFY* genes were inferred as promiscuous, so the ancestral state of all embryophyte *LFY* (including type I, type II, and promiscuous genes) were also recognized as the promiscuous form (Fig. 3).

### Genomic synteny conservation and transposition in grasses

Genomic synteny is common among angiosperms and flowering genes such as *MADS-Box*^47^ and *VOZ*^33^ that are located in conserved genomic regions. In light of this, we looked for genomic syntenies around the *LFY* loci, making use of the available plant genomes deposited in the Phytozome database.

**Fig. 4:**
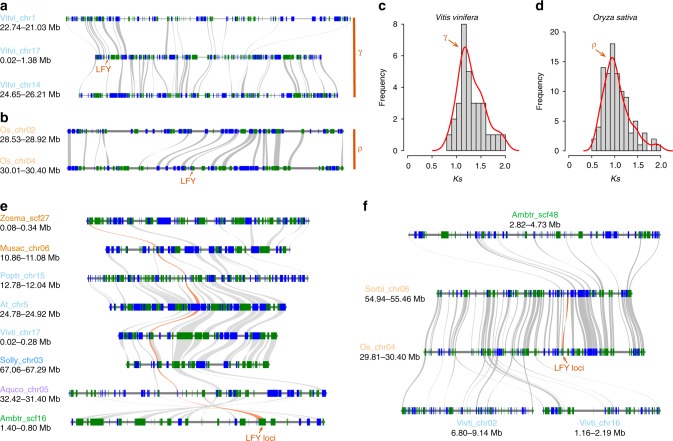
Collinearity of the *LFY-*associated genomic blocks in representative angiosperm genomes. **a** Neighboring genomic region of the *LFY* locus in the grapevine (*Vitis vinifera*) genome is syntenic to another two genomic blocks without *LFY* genes, which together constituted the gamma triplicated genomic blocks. **b** Neighboring genomic region of the *LFY* locus in rice (*Oryza sativa*) genome is syntenic to another genomic blocks without *LFY* gene, which is congruent to the rho event. **c**, **d** Synonymous substitutions (*Ks*) distributions of the *LFY* loci neighboring syntelogs in rice and grapevine genomic blocks (Supplementary Data 6), providing molecular dating evidences supporting the ancestral polyploidy events. **e** Multiple genomic synteny alignments of the *LFY-*associated genomic blocks in representative species, including two monocots (*Zostera marina* and *Musa acuminata*), three rosids (*Populus trichocarpa*, *Arabidopsis thaliana*, and *Vitis vinifera*), one asterid (*Solanum lycopersicum*), the basal eudicot (*Aquilegia coerulea*), and the basal angiosperm (*Amborella trichopoda*). The syntenic connections of the *LFY* gene loci were highlighted with orange strips. **f** The transposed *LFY* genes in the grasses rice (*Oryza sativa*) and sorghum (*Sorghum bicolor*) were located in very conserved syntenic genomic regions, but the neighboring regions are syntenic to genomic regions not associated with *LFY* genes in grapevine and *Amborella* genomes. The plant species were depicted as their abbreviated five-letter scientific names, the chromosome/scaffold numbers and genomic block coordinates were also included. Genes on forward and reverse strands were depicted as blue and green blocks, respectively

To assess the loss of *LFY* genes during land plant evolution, the genomic synteny of adjacent genes of *LFY* loci were investigated in the genomes of grape (*Vitis vinifera*) and rice (*Oryza sativa*), whose genomes were well characterized in relation to the genome triplication event shared by core eudicots (the gamma event, ~117 Mya)^48,49^ and the grass-wide genome duplication (the rho event, ~66 Mya)^50^, respectively. In the grape genome, the single-copy *LFY* gene was found in chromosome 17, but its adjacent genomic region was syntenic to another two genomic blocks in chromosome 1 and chromosome 14 (Fig. 4a), which together represent the three subgenomes of the gamma event^48^. The synonymous substitution analyses of the associated syntelogs revealed a conspicuous *Ks* peak around 1.17 (Fig. 4c; Supplementary Data 6), consistent with the gamma peak in the grape genome^49^. Similarly, the *LFY* gene is located in chromosome 4 of the rice genome, and a large syntenic genomic counterpart was found in chromosome 2 where the *LFY* locus was absent (Fig. 4b). The syntelog pairs found in the large genomic collinear region demonstrated a conspicuous *Ks* peak around 0.95 (Fig. 4d; Supplementary Data 6), which is consistent with the divergence level of paralogues derived from the rho event^51^. The identification of collinear genomic regions where *LFY* genes were lost suggested *LFY* genes were restored to a single copy in most angiosperm genomes, probably by gene-specific losses, instead of the removal of large genomic blocks, at least for the gamma and rho events in core eudicots and grasses, respectively.

While retention as a single copy in most angiosperms, the *LFY* loci were also located in highly conserved genomic regions, as determined by aligning the neighboring genomic syntenic blocks (Fig. 4e). The results demonstrate that genomic regions containing *LFY* genes from representative plant genomes, including monocots (*Zostera marina* and *Musa acuminata*), rosids (*Populus tricoporda*, *Arabidopsis thaliana* and *Vitis vinifera*), asterids (*Solanum lycopersicum*), basal eudicot (*Aquilegia coerulea*), and the basal-most angiosperm *Amborella trichopoda*, were clustered by syntenic connections. However, the genomic context of the *LFY* loci from rice and sorghum were different from those of the plant lineages mentioned above. In these instances, the neighboring genomic region was syntenic to non-*LFY* genomic regions in *Vitis* and *Amborella* (Fig. 4f), suggesting an ancestral translocation of *LFY* genes in grasses.

**Fig. 5:**
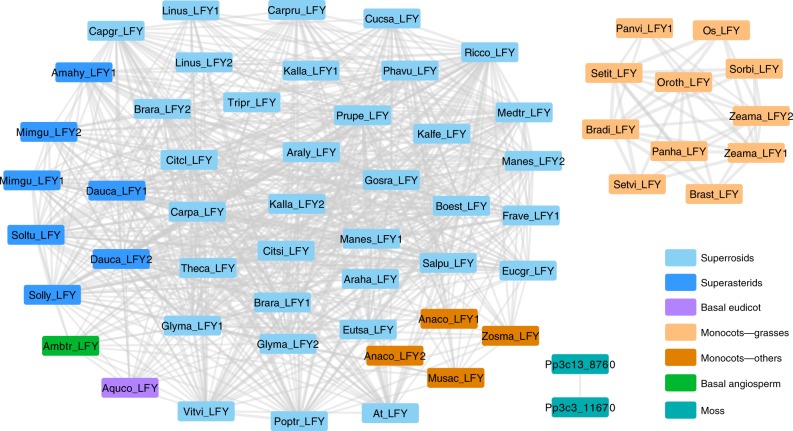
Genomic synteny network of LFY genes among plant genomes. The syntenic network among plant genomes was constructed using the LFY gene loci as anchors. Species in the network were represented by five-letter abbreviations (Supplementary Data 1). Pairs of LFY genes were connected by lines if identified in the corresponding genomic syntenic regions. Line weights are in proportion to the syntenic block score (log transformed) calculated by MCScanX (Supplementary Data 7), where thicker lines largely indicate larger syntenic genomic blocks containing more syntelogs

To further investigate the genomic syntenies of *LFY* loci, all the *LFY-*associated genomic synteny blocks were extracted to construct the comprehensive genomic synteny network, where the nodes in the network represented the *LFY*-associated genomic regions and edges connecting nodes representing syntenic relationships (Fig. 5; Supplementary Data 7). The two moss *LFY* genes were found to be located in syntenic regions in the *P. patens* genome, consistent to its recent whole-genome duplication^52^ and were separated from all other angiosperms as a result of the long divergence time. Unlike the *VOZ* gene family that were conserved among angiosperms^33^, two isolated synteny networks for *LFY* were obtained: one containing angiosperm *LFY* genes including eudicots, monocots, and the basal angiosperm *Amborella*, the other containing genes specifically from grasses. *LFY* genes identified from pineapple, banana, seagrass, and *Amborella* genomes were all clustered with the eudicot genes, thus this comprehensive genomic synteny comparison suggests an ancestral gene transposition occurred in the ancestor of grasses. The gene structure of *LFY* from grasses and eudicots is well conserved with three coding regions (Supplementary Fig. 16), suggesting the gene structure was not altered by the ancestral transposition event. Moreover, among the genomes of grasses, the *LFY* loci-associated genomic collinearities were highly conserved with relatively larger and more conserved synteny blocks (Fig. 4f and thicker edges in Fig. 5).

## Discussion

Our analyses lead to the conclusion that the *Osmunda* promiscuous *LFY* genes are products of an ancient transient duplication. Nevertheless, the possibility of ancient symbiotic or gene transfers from hornworts to *Osmunda* cannot be entirely eliminated. However, neither maximum-likelihood nor Bayesian phylogenies supports the monophyly of promiscuous *LFY* genes from hornworts and *Osmunda* with high confidence (posterior probability <50% in Bayesian analyses), suggesting their distant phylogenetic relationships, which makes sequence contaminations from hornworts highly unlikely. We propose to delineate the promiscuous genes identified from *Osmunda* as soloist genes, as these genes, derived from the proposed ancient transient duplication, are likely to be very rare.

While the family phylogeny confidently supports an ancient gene duplication before the divergence of type I and type II genes, we propose this duplication event would be better supported by the identification of type II genes from tracheophytes in future studies when more genomic sequences become available, but it is also possible that the type II *LFY* paralogues were all lost in extant tracheophytes. Furthermore, current large-scale phylogenomic studies^19,46^ have not detected an ancient polyploidy event in the ancestor of mosses, liverworts, and tracheophytes, so this ancient gene duplication was likely derived from a small-scale duplication (e.g., segmental or tandem duplication) event. The type I and type II duplication hypothesis can be questioned, however, if the Bryophytes-monophyletic land plant phylogeny, where hornworts, liverworts, and mosses constituted the monophyletic clade that is sister to tracheophytes (Fig. 2b), is employed. In this scenario, the *LFY* members were diversified within bryophytes, where all the promiscuous, type I and type II *LFY* genes evolved and were retained, and only type I genes were fixed in the tracheophytes. However, this hypothesis can be rejected as all type I genes (across taxa) are monophyletic, with high confidence nodal bootstrap support.

The ancestral state reconstruction analyses suggested type I and type II genes were derived from promiscuous genes through a duplication and sub-functionalization process. The *LFY* family phylogeny we generated also amended the tree topology proposed by Brunkard et al.^30^ where the type I and type II paralogues from the moss *Polytrichum commune* were incorrectly placed as close sisters, as if they were derived from a moss-specific gene duplication. Moreover, this ancestral state analyses re-confirmed that the promiscuous *LFY* intermediate form did overlay the water-to-land transition process because the ancestral state of embryophyte gene was reconstructed as promiscuous. However, transient duplications may have occurred in the evolutionary past of the of the *LFY* genes and left soloist genes in *Osmunda*.

Overall, gene tree uncertainty and the reservations in the proposed land plant phylogeny, even though only two phylogenies (Fig. 2a, b) are currently under consideration, make insights into the early evolution of *LFY* genes more elusive. Nevertheless, the robust phylogenetic analyses with augmented sequence data sources that we present support the hypothesis that the type I and type II *LFY* genes were products of an ancient gene duplication and sub-functionalization from the promiscuous gene. Furthermore, the identification of promiscuous genes from ferns also suggested that the promiscuous intermediates experienced an ancient embryophyte-transient duplication event. These observations taken together lead us to suggest that both promiscuous transition and gene duplication followed by sub-functionalization were involved in the evolutionary past of this important transcription factor family.

Although the induction and fixation processes that generated the ancestral grass-specific transposition remain unknown, the gene transposition could have introduced *cis-*regulatory elements and new chromatin interactions for the *LFY* loci in the new genomic context. The new genomic context in turn explains the observed changes in the gene expression patterns for *LFY* and *RFL* genes with *LFY* in *Arabidopsis* expressed uniformly in floral meristem (Supplementary Figs 10, 11) instead of apical inflorescence meristem^3^, and *RFL* in rice demonstrated high expression level in young panicles including the apical meristem (Supplementary Fig. 13), but expressed minimally in the floral meristem^9^. However, the expression pattern of *RFL* is also distinct from the two *LFY* homologs in maize (*ZFL1* and *ZFL2)*, which are highly expressed in branching spikelet meristems and floret meristems instead of the inflorescence apex^53^. The distinctive expressions of *LFY* homologs suggests differentiated regulatory mechanisms not only arose from the translocation activity but also as a consequence of the length of the time of divergence after speciation. Grasses also exhibit highly divergent floral morphology with multiple kinds of branch meristems. Grasses do not have clear homologs to the sepals and petals of typical eudicots, so the developmental models established in Arabidopsis may not be completely applicable to other remote lineages, which diverged from each other more than 150 Mya: the highly diverged gene functions and expressions of grass *LFY* homologs would represent such an example. Some other grass-specific transpositions such as the regulators of root development (*AGL17*) were also reported and might be associated with the emergence of lineage-specific regulatory mechanisms accompanied with the alteration of genomic context (i.e., translocation)^47^. One possible lineage-specific regulatory mechanism could involve lineage-specific miRNAs and transposition of target genes, which might constitute a lineage-specific regulatory network^47^ and facilitate the diversification process.

In conclusion, as a supplement and reassessment of the gene duplication hypothesis proposed by Brunkard et al.^30^, our comprehensive phylogenetic analyses provided strong evidence that the type I and type II *LFY* genes were derived from sub-functionalization of promiscuous genes after an ancient duplication event shared by mosses, liverworts, and tracheophytes. The phylogenetic placement of this type I and type II duplication could be elusive because of uncertainty in the land plant phylogeny and the absence of type II genes in tracheophytes. The identification of promiscuous *LFY* from ferns (i.e., the basal lineage *Osmunda*) implied that the promiscuous *LFY* has experienced an ancient embryophyte duplication, which was transient and promptly lost in most extant land plant lineages. We suggested that the early evolution of *LFY* still complies with the duplication and sub-/neo-functionalization evolutionary regime, with the promiscuous intermediate form smoothing the process of the conquest of land by plants. The augmented genomic synteny comparisons revealed the genomic relics that remain after gene losses, and the comprehensive synteny network revealed the ancestral grass-specific transposition activity in the evolutionary history of this focal transcription factor. We believe that as more plant genomes accumulated they will provide us the resources needed to make new discoveries in a more comprehensive and robust manner. We also recommend that future gene family evolution studies should be placed into the framework of plant diversity so that information losses are minimized.

## Methods

### Mining genomes and transcriptomes for LFY homologs

The homologs of plant *LFY* transcription factor genes (Supplementary Data 1) were collected from Phytozome v12.1.6 (https://phytozome.jgi.doe.gov/pz/portal.html) and the OneKP (https://db.cngb.org/onekp/)^54^ databases using blastp searches and filtered with an e-value threshold of 1e-5. Specially, the *LFY* gene sequences in cucurbits and ferns were collected from the Cucurbit Genomics Database (CuGenDB, http://cucurbitgenomics.org)^55^ and FernBase (https://www.fernbase.org)^56^, respectively. The protein domain compositions of each of the putative LFY protein sequences were determined by querying the NCBI Conserved Domain Database^57^ and only the sequences that contained an intact FLO_LFY domain were included in our subsequent phylogenetic analyses (Supplementary Data 2). The functional domains were queried from the Pfam database^58^, and sequence motif patterns were analyzed using the MEME suite^59^, and motif patterns were plotted using the TBtools (https://github.com/CJ-Chen/TBtools).

### Family phylogeny reconstruction

Generating a reliable sequence alignment for the *LFY* members was crucial for accurate gene family phylogeny reconstruction. Based on the sequence signature of the LFY protein sequences, we identified the sequence boundaries of the SAM_LFY (PF01698.16) and C_LFY_FLO (PF17538.2) domains by aligning each of the protein sequences onto the two HMM profiles using hmmalign v3.1b2^60,61^. Alignments of the two signature domains were aligned separately and concatenated, columns in the alignment with less than 20% sequence occupancy were removed using Phyutility v2.2.6^62^.

The IQ-TREE v1.6.8^63^ program was employed to reconstruct the maximum-likelihood gene tree. For the obtained broad-scale amino acid alignment (Supplementary Data 3), JTT + G + I was the best-fitting evolutionary model selected by ModelFinder^64^ under Bayesian Information Criterion, the SH-aLRT test and ultrafast bootstrap^65^ with 1000 replicates were conducted in IQ-TREE to obtain the supporting values for each internal node of the tree (Supplementary Data 4). Bayesian inference phylogenetic analyses were performed using Mrbayes v3.2.6^66^ with 11 million generations, with trees sampled every 1000 generations. The first 25% of the sampled trees were discarded as burn-in and the remaining were used to generate the consensus tree and calculate the Bayesian posterior probabilities. To ensure the Bayesian MCMC runs were sufficient to reach convergence, Tracer v1.7.1^67^ (http://tree.bio.ed.ac.uk/software/tracer/) was employed to analyze the trace files to ensure the Effective Sample Size was larger than 200 and the Potential Scale Reduction Factor was very close to one (Supplementary Data 5). The obtained maximum-likelihood and Bayesian gene trees were visualized and edited using FigTree v1.4.4 (http://tree.bio.ed.ac.uk/software/figtree/).

### Ancestral state inference

Considering the uncertainty of the phylogenetic position of the promiscuous genes from *Osmunda*, the two alternative collapsed tree topologies generated by maximum-likelihood and Bayesian analyses were assumed in the ancestral state reconstruction. The ancestral state of the three focal amino acids in embryophytes were inferred using the maximum-likelihood algorithm implemented in MEGA v7.0.26^68^.

### Genomic synteny comparison and network construction

To analyze the genomic synteny relationships among plants, protein sequences for each of the angiosperm genomes from the Phytozome v12.1.6 database were compared to each other using the Diamond v0.9.22.123 program^69^ with an e-value cutoff at 1e-5. Only the top five non-self blastp hits were retained as input for MCScanX^70^ analyses. The genomic synteny plots for the *LFY* loci-associated chromosomal regions were generated using the Python JCVI utilities developed by Haibao Tang and colleagues (https://github.com/tanghaibao/jcvi). Protein sequences for the syntelog pairs were aligned using Muscle v3.8.31^71^ and translated back to coding sequence alignments using the perl script PAL2NAL v14^72^, then synonymous substitution rates (i.e., *Ks* or *Ds*) for syntelogs were calculated using the KaKs_calculator v2.0^73^ using the Goldman & Yang (-m GY) model (Supplementary Data 6). The rice syntelog pairs with average GC content higher than 75% at the third positions of codons were unreliable and discarded^51^. The peak of the obtained *Ks* values within the range of (0,2] were analyzed using kernel density estimation function in R statistical environment. To generate the comprehensive synteny network, all the *LFY* gene anchored syntenic genomic block were extracted (Supplementary Data 7) and visualized in Cytoscape v3.7.0^74^. It should be noted that some truncated *LFY* loci that does not contain the intact signature domains could be detected as syntenic to intact *LFY* genes. The thickness of edges in the synteny network were depicted based on the syntenic block score (log transformed) calculated by MCScanX.

### Reporting summary

Further information on research design is available in the Nature Research Reporting Summary linked to this article.

## Supplementary information

Supplementary material

Descriptions of Supplementary Data

Supplementary Data 1

Supplementary Data 2

Supplementary Data 3

Supplementary Data 4

Supplementary Data 5

Supplementary Data 6

Supplementary Data 7

Reporting Summary

## Data Availability

All data supporting the findings of this study are available within the published article (and its Supplementary Information files).
