# Supplementary material for: Ancient duplications and grass-specific transposition influenced the evolution of LEAFY transcription factor genes
Source: Commun Biol. 2019 Jun 21;2:237. doi: 10.1038/s42003-019-0469-4 (PMC6588583; doi:10.1038/s42003-019-0469-4)
Supplement: Supplementary file 1 — Supplementary material [file 42003_2019_469_MOESM1_ESM.pdf]

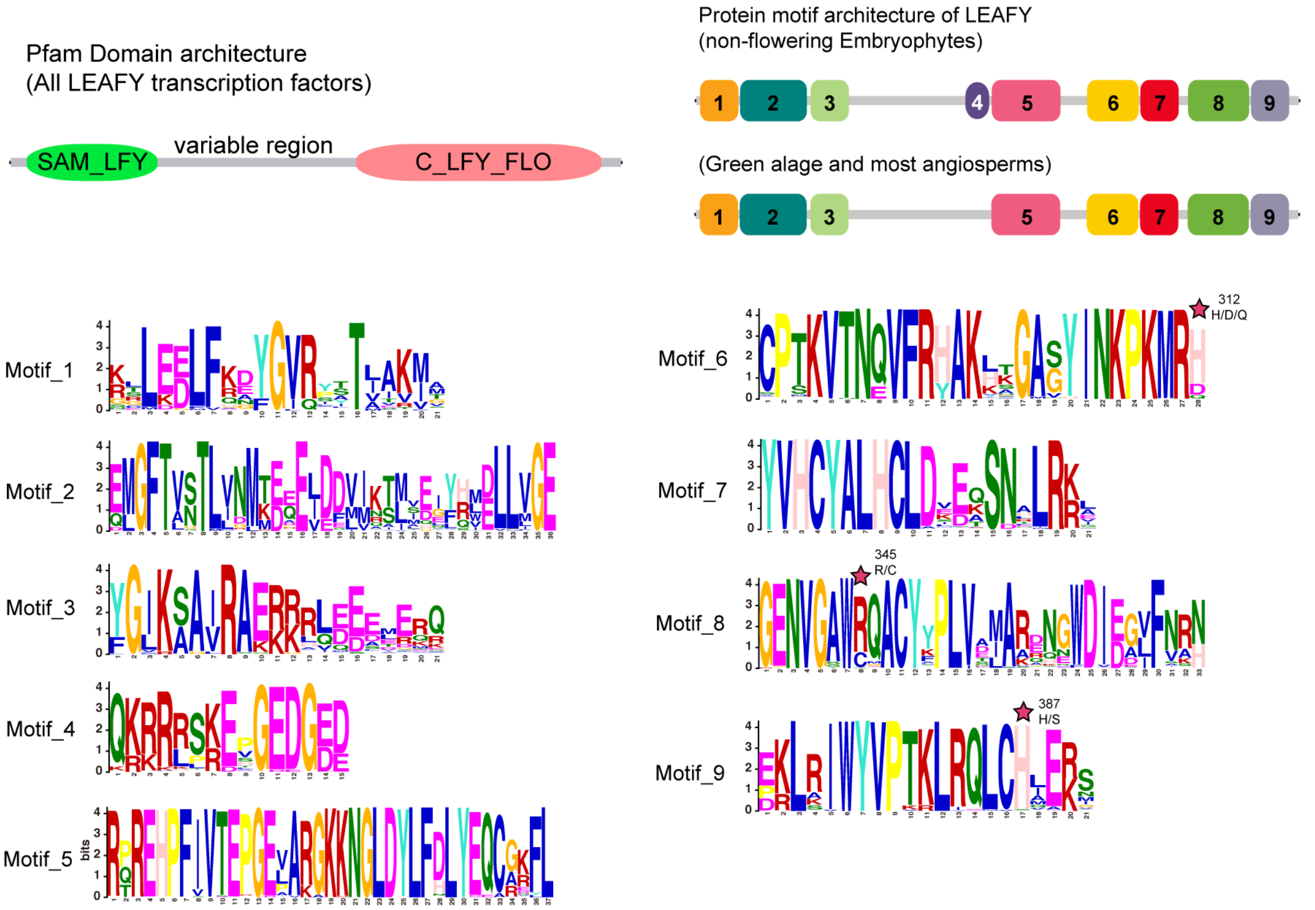

Supplementary Figure 1. Characteristics of the *LEAFY* transcription factor sequences. All LEAFY transcription factors contain a SAM_LFY (PF01698.16) domain at the N terminal region and the DNA-binding domain C_LFY_FLO (PF17538.2) at the C terminal region. MEME protein sequence motif analyses recaptured the two conserved domains. Sequence logo for each motif (motif_1 through motif_9) were depicted and the three critical amino acid sites (312, 345 and 387) in motif_6, motif_8 and motif_9 were also labeled with stars.

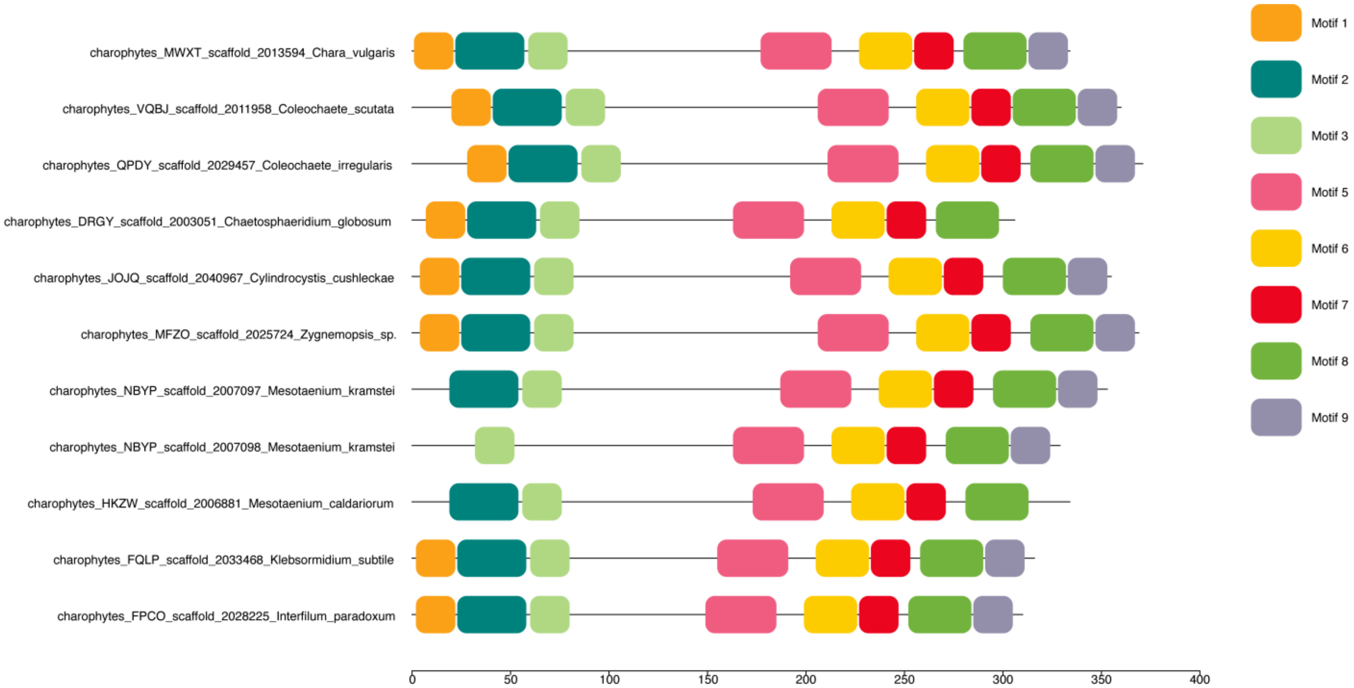

Supplementary Figure 2. Protein sequence motif analyses of *LEAFY* homologs in charophytes. Sequence logos for each motif were depicted in Supplementary Figure 1.

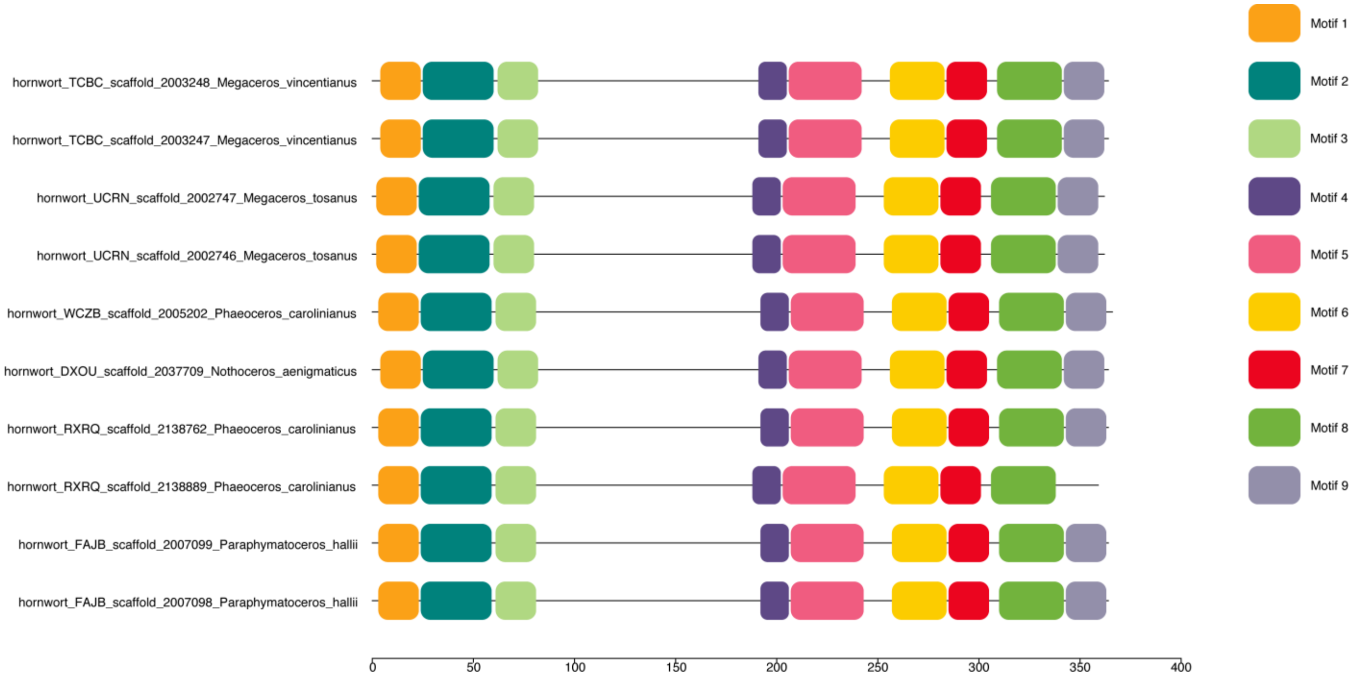

Supplementary Figure 3. Protein sequence motif analyses of *LEAFY* homologs in hornworts. Sequence logos for each motif were depicted in Supplementary Figure 1.

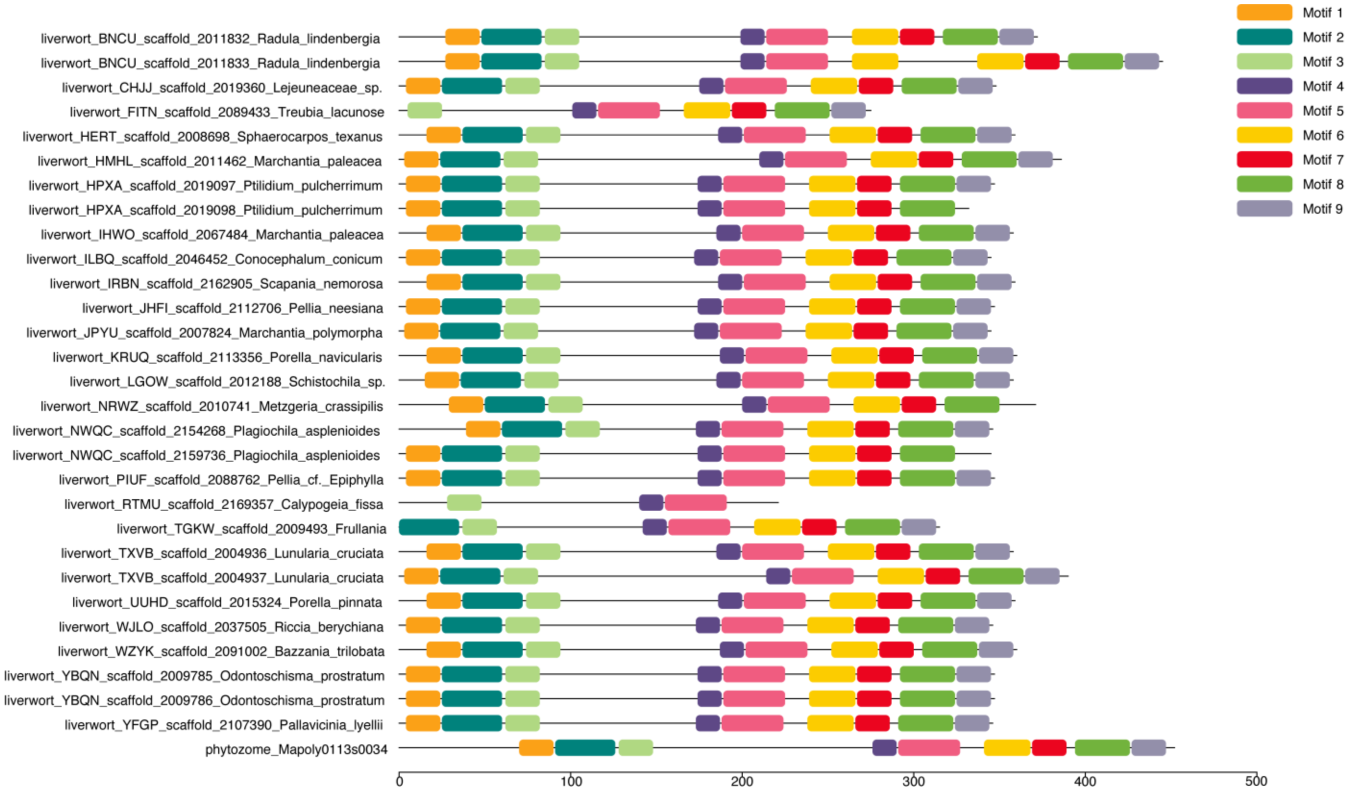

Supplementary Figure 4. Protein sequence motif analyses of *LEAFY* homologs in liverworts. Sequence logos for each motif were depicted in Supplementary Figure 1.

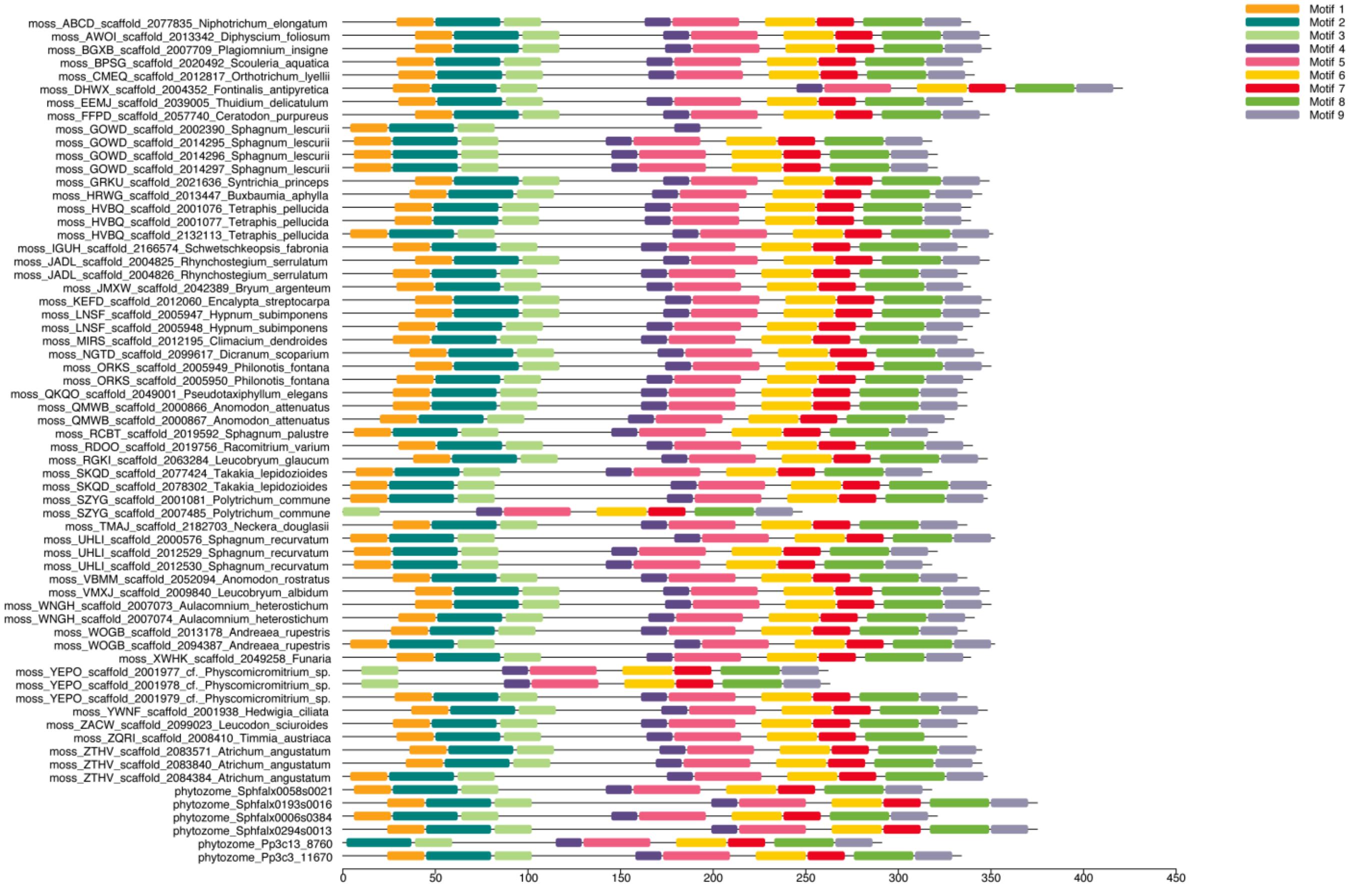

Supplementary Figure 5. Protein sequence motif analyses of *LEAFY* homologs in mosses. Sequence logos for each motif were depicted in Supplementary Figure 1.

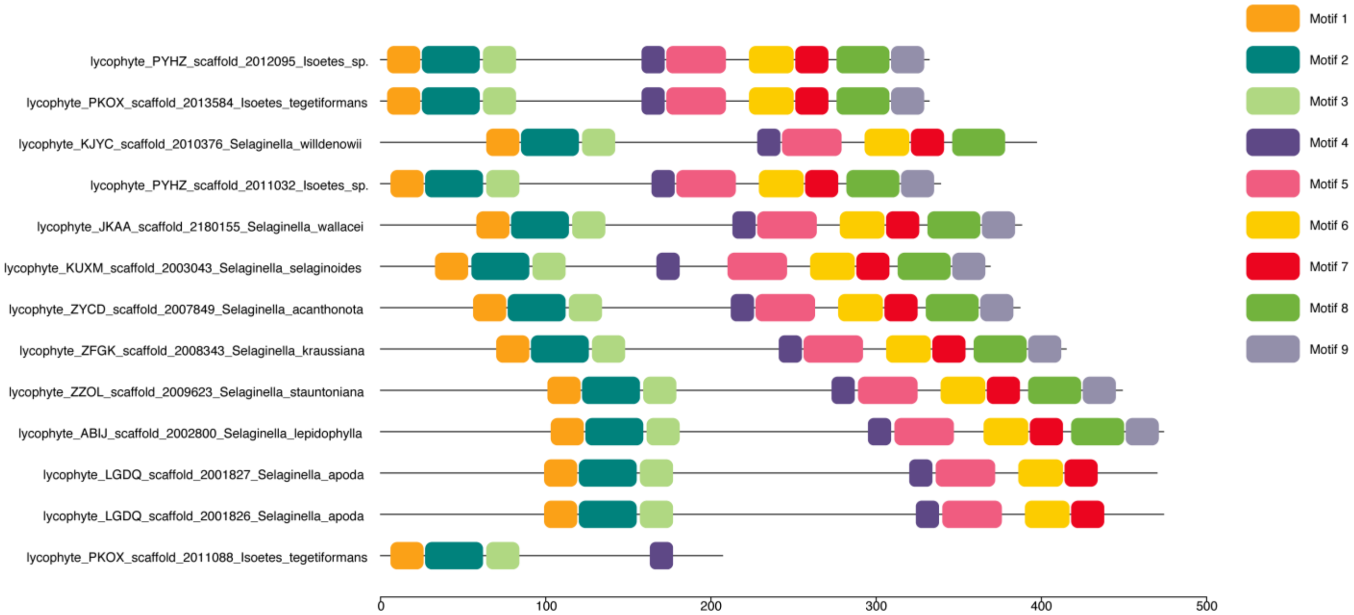

Supplementary Figure 6. Protein sequence motif analyses of *LEAFY* homologs in lycophytes. Sequence logos for each motif were depicted in Supplementary Figure 1.

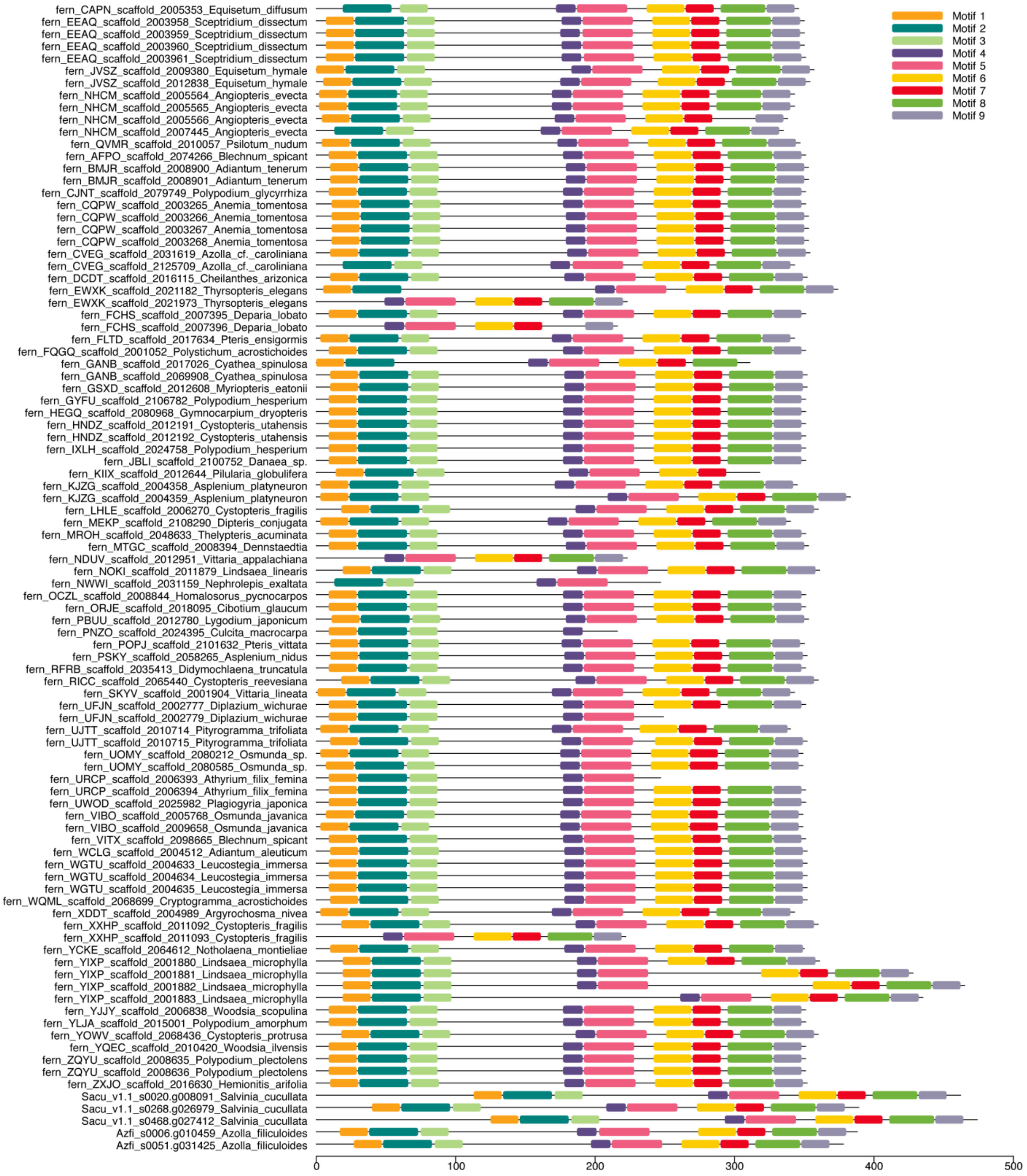

Supplementary Figure S7. Protein sequence motif analyses of *LEAFY* homologs in ferns. Sequence logos for each motif were depicted in Supplementary Figure 1.

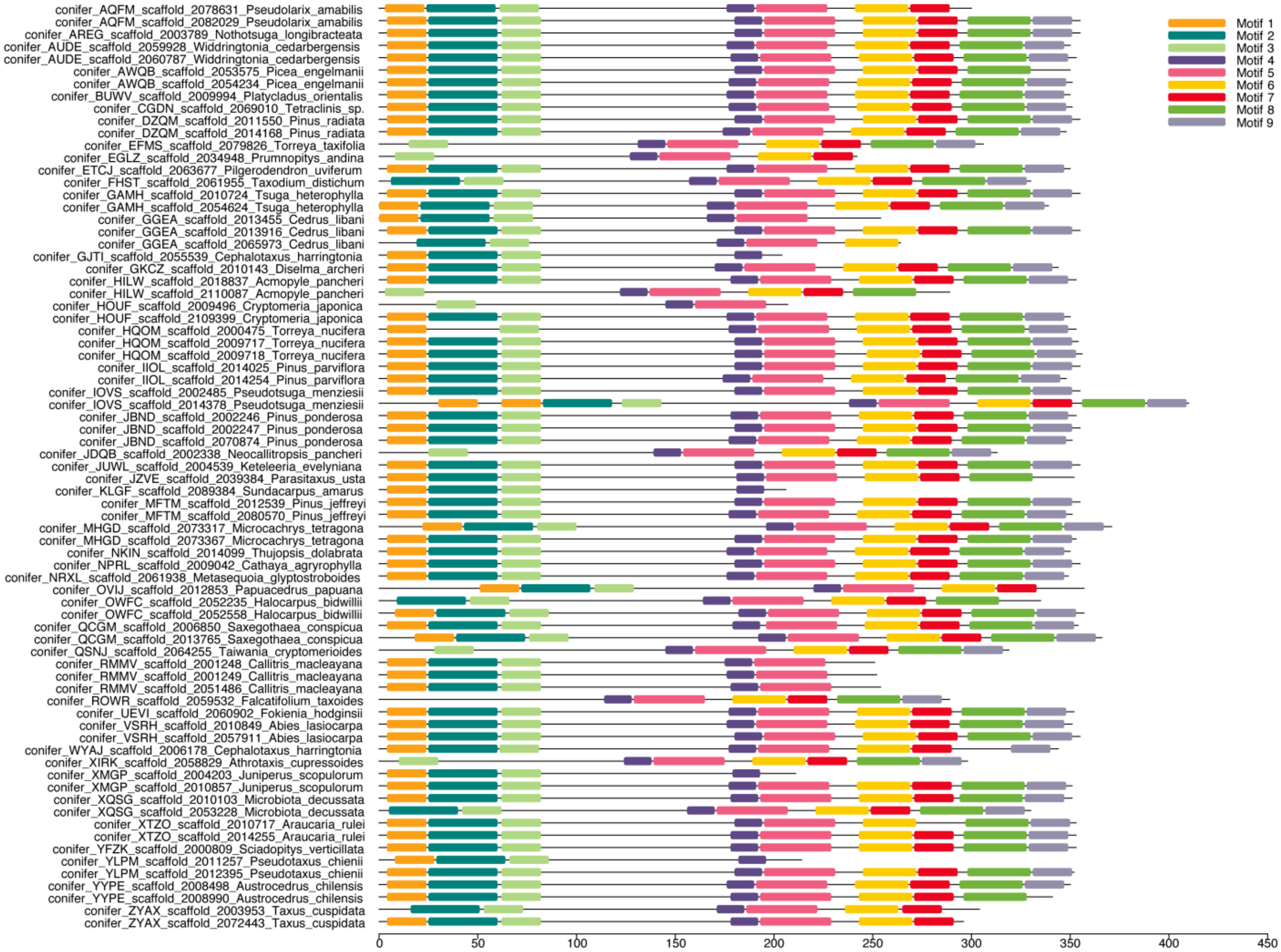

Supplementary Figure 8. Protein sequence motif analyses of *LEAFY* homologs in conifers. Sequence logos for each motif were depicted in Supplementary Figure 1.

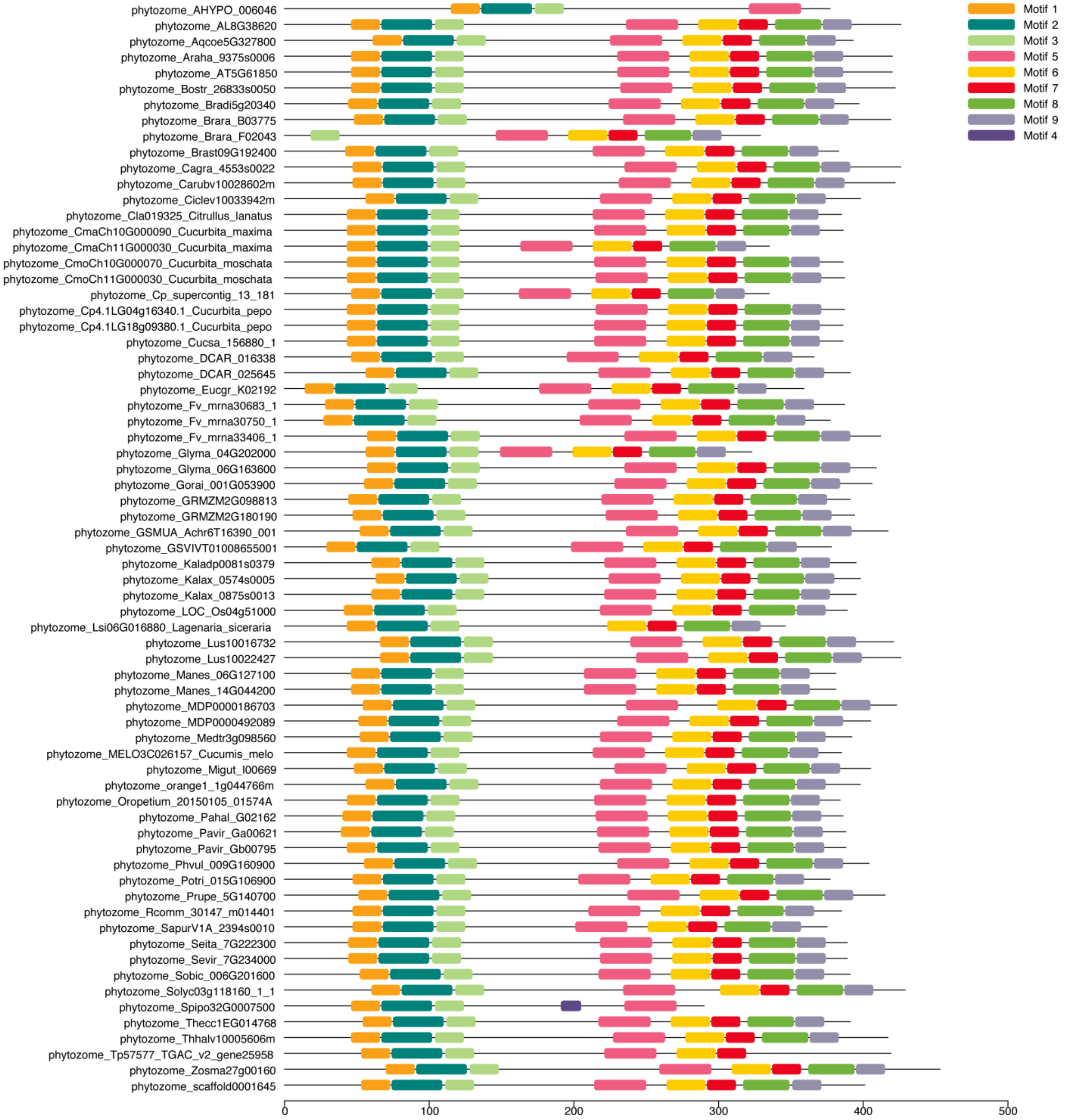

Supplementary Figure 9. Protein sequence motif analyses of *LEAFY* homologs in angiosperms. Sequence logos for each motif were depicted in Supplementary Figure 1.

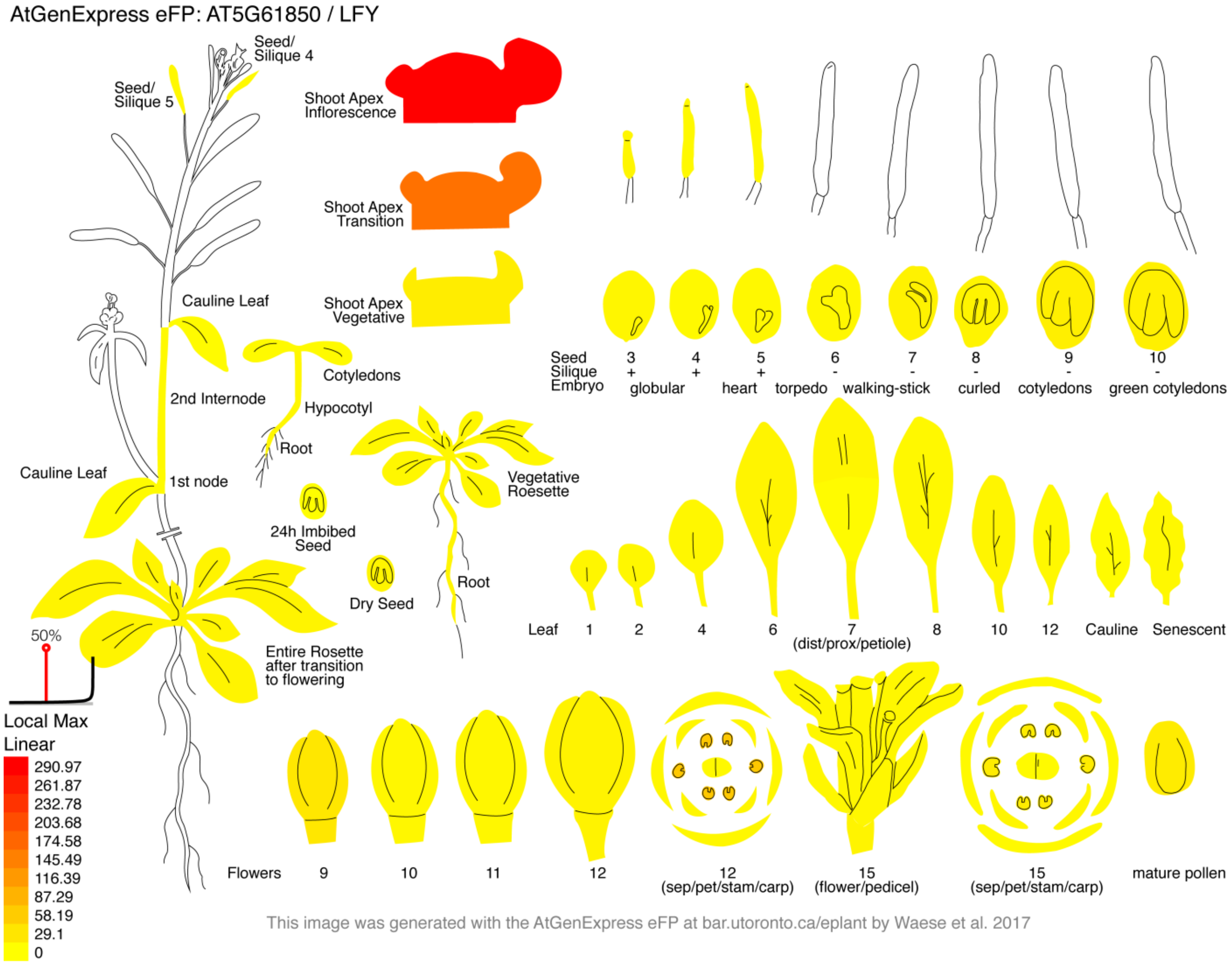

Supplementary Figure 10. Gene expression profile of *LEAFY* in *Arabidopsis thaliana* tissues. Image was generated in the AtGenExpress database and could be retrieved from http://bar.utoronto.ca/eplant/.

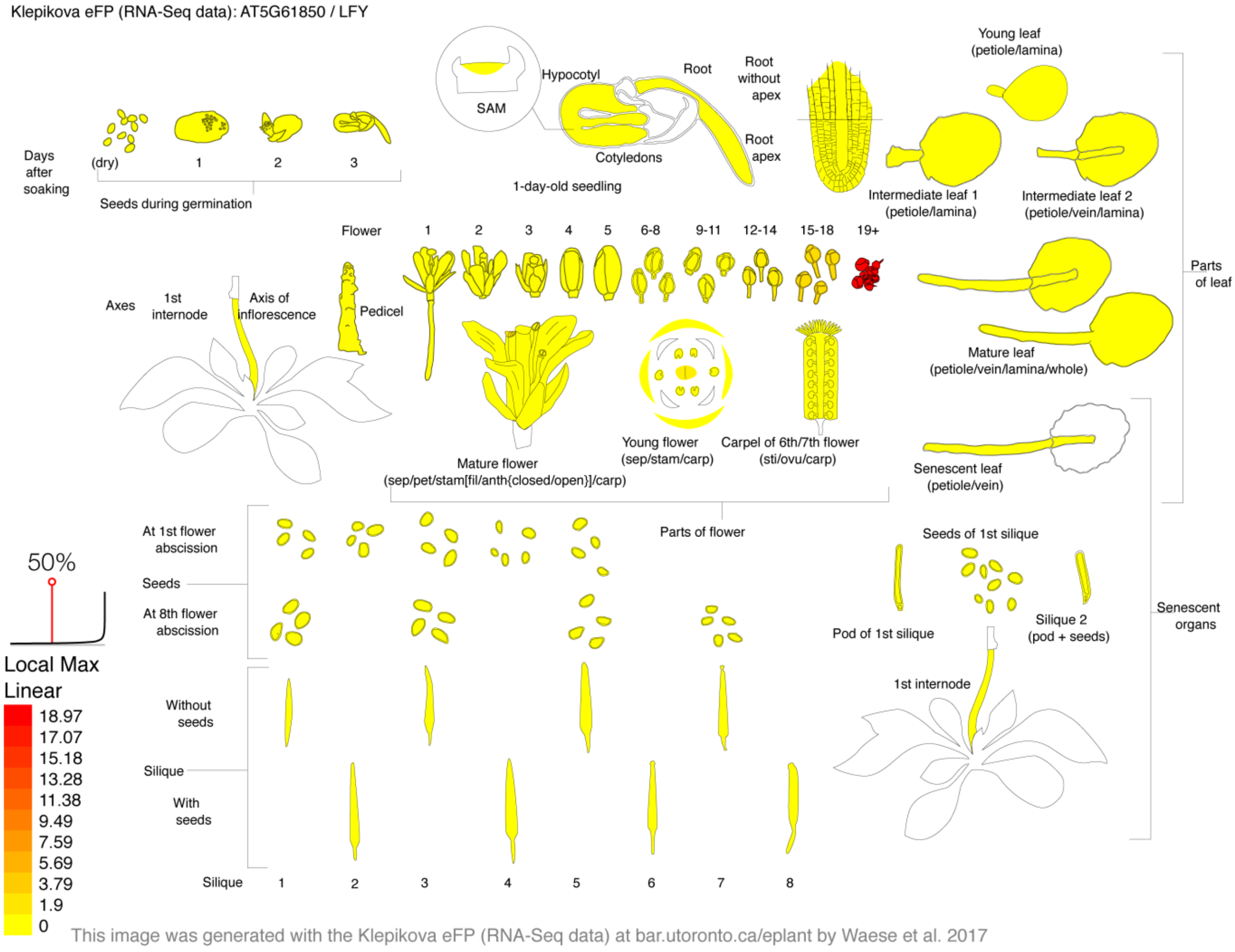

Supplementary Figure 11. Gene expression profile of *LEAFY* in *Arabidopsis thaliana* tissues. Data and image were from the Klepikova eFP (RNA-Seq data) database and could be retrieved in http://bar.utoronto.ca/eplant/.

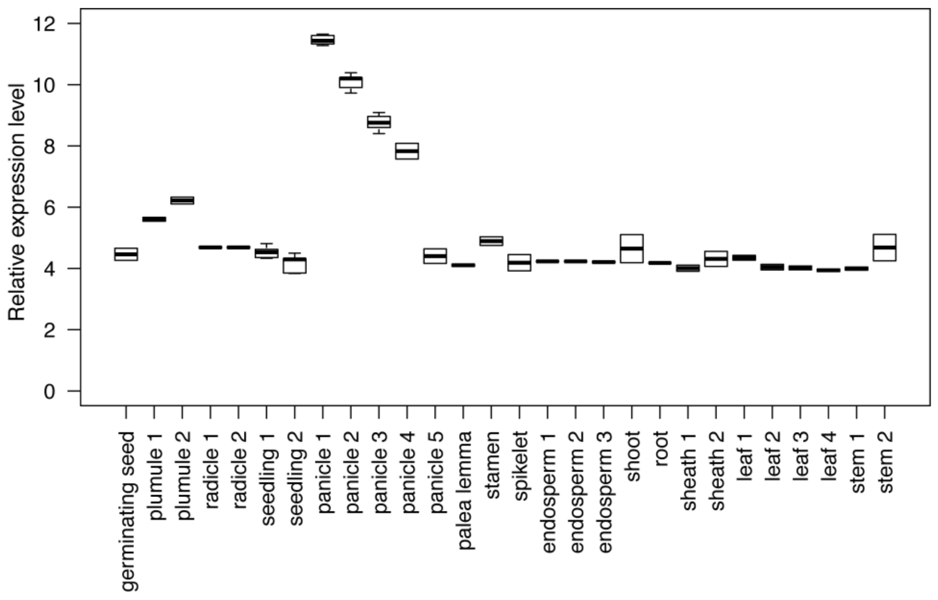

Supplementary Figure 12. Gene expression profile of *LEAFY* homolog in *Oryza sativa* tissues. Gene expression data (NCBI GEO accession: GSE19024) were originally from developmental transcriptomes in Minghui 63 rice (Wang *et al.*, 2010; PMID: 20003165).

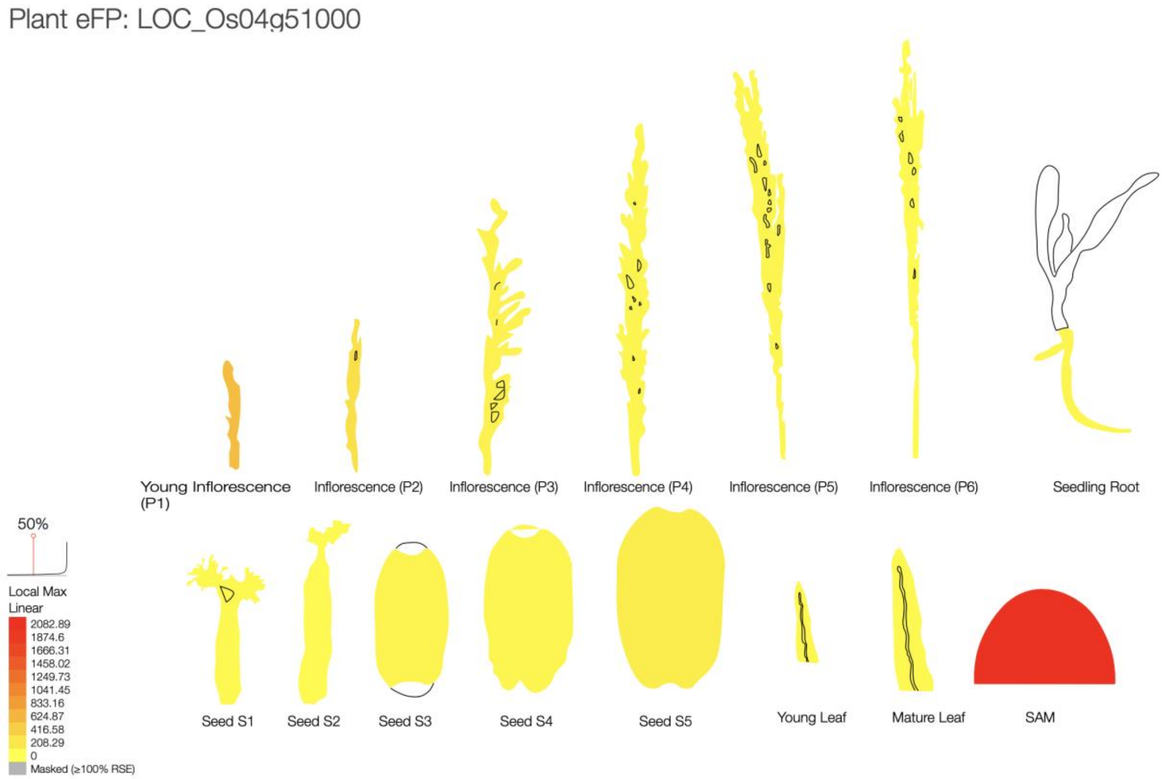

Supplementary Figure 13. Gene expression profile of *LEAFY homolog* in *Oryza sativa* tissues. Data and image were obtained from the Rice eFP Browser (http://bar.utoronto.ca/eplant_rice/).

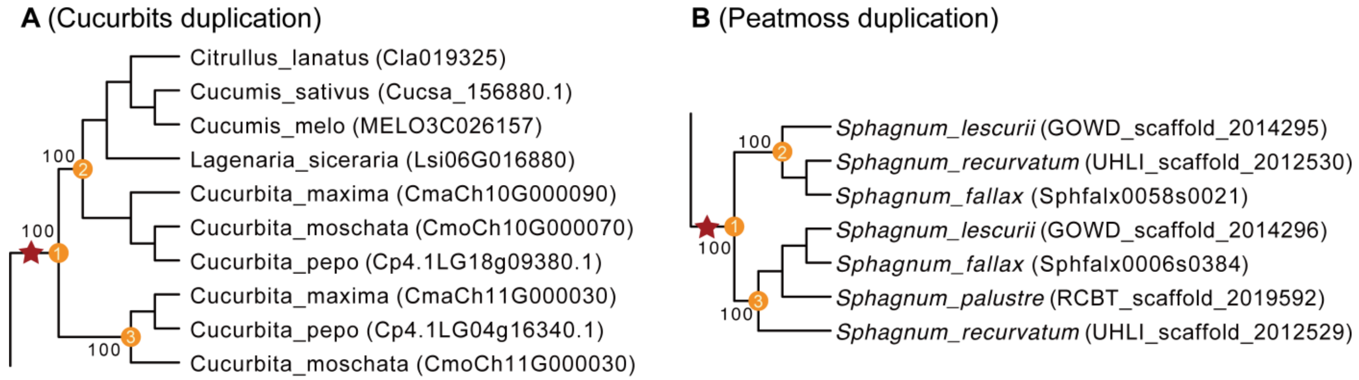

Supplementary Figure 14. The cucurbits duplication and peat-moss duplication were captured in the LEAFY family phylogeny. Corresponding bootstrap supporting values for the three nodes supporting the duplication were indicated and duplication modes were labeled with a red star.

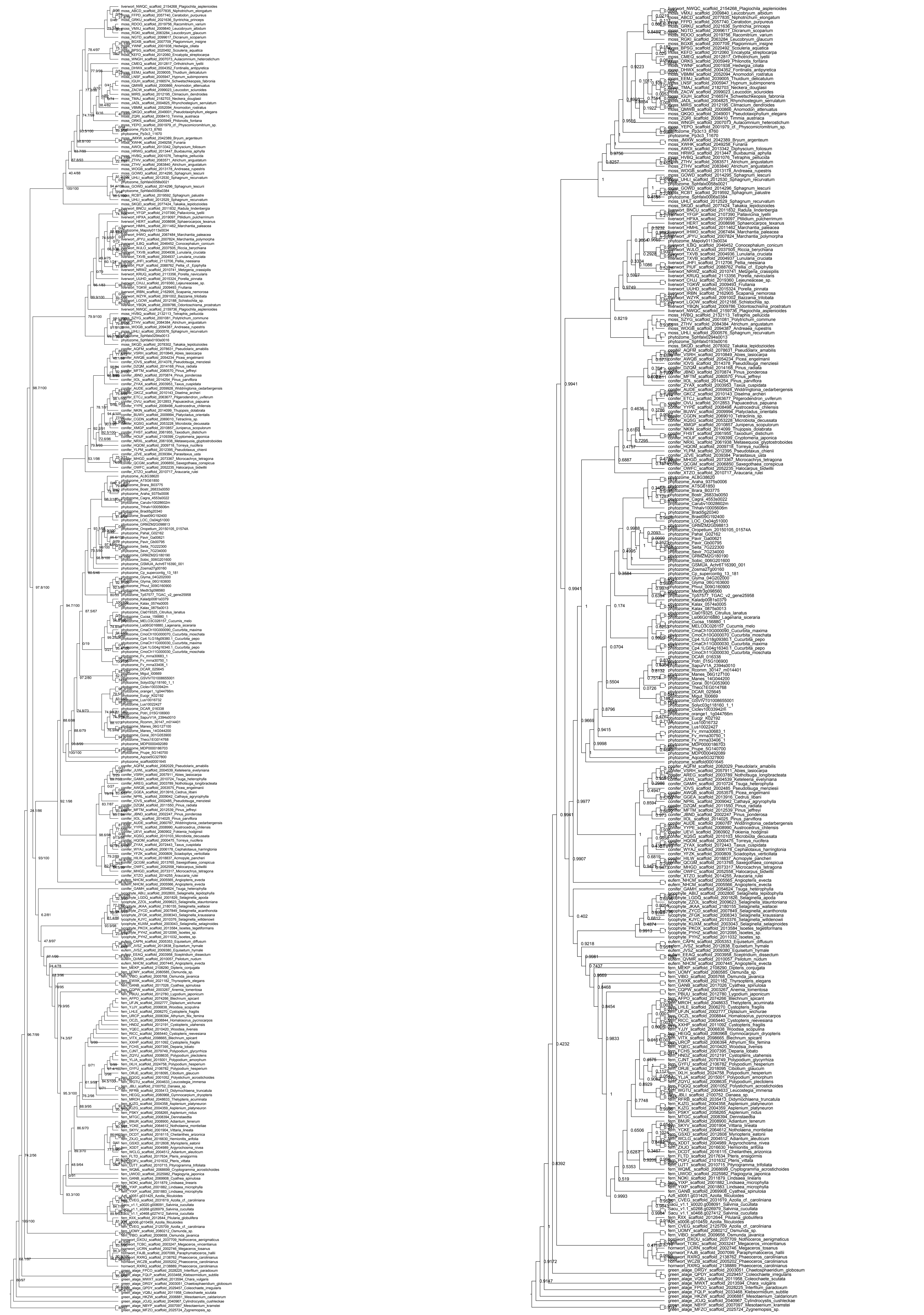

Supplementary Figure 15. The LEAFY gene family phylogeny reconstructed using maximum-likelihood (ML, left panel) and Bayesian inference (BI, right panel) approaches. The SH-aLRT test/bootstrap values supporting the nodes were labeled in the ML tree and posterior probabilities were labeled in the BI tree.

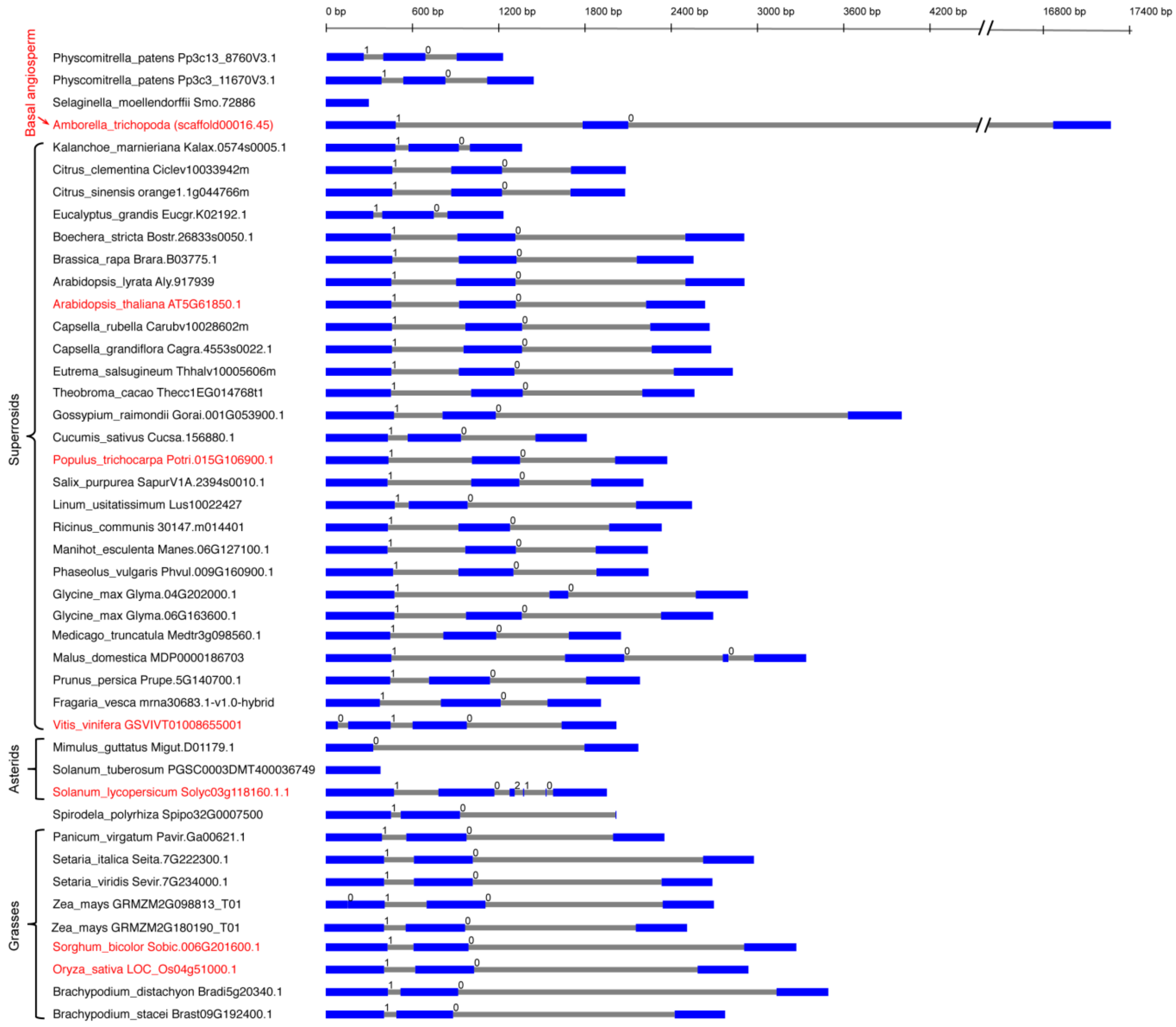

Supplementary Figure 16. Patterns of coding region and intron structures of LEAFY homologous genes in plants. The coding regions of each gene were plotted as blue boxes and introns as grey lines.
