# Supplementary material for: Ancient duplications and grass-specific transposition influenced the evolution of LEAFY transcription factor genes
Source: Commun Biol. 2019 Jun 21;2:237. doi: 10.1038/s42003-019-0469-4 (PMC6588583; doi:10.1038/s42003-019-0469-4)
Supplement: Supplementary file 2 — Descriptions of Supplementary Data [file 42003_2019_469_MOESM2_ESM.docx]

**Description of additional supplementary files**

**File Name:** Supplementary Data 1

**Description:** Lists of identified LEAFY transcription factor genes in each of the plant lineages.

**File Name:** Supplementary Data 2

**Description:** The raw protein sequences of identified LEAFY homologs. The names of the sequences in FASTA format were edited to include the taxonomy information.

**File Name:** Supplementary Data 3

**Description:** Protein sequence alignments of LEAFY family. The alignments were generated by concatenating the SAM_LFY (PF01698.16) and C_LFY_FLO (PF17538.2) domains. This sequence alignment was utilized to reconstruct the family phylogeny. The alignments depicted in figure 1 were derived from this global alignment.

**File Name:** Supplementary Data 4

**Description:** The maximum-likelihood gene family tree. The phylogenic tree was transformed as cladogram in NEXUS format and could be visualized using the FigureTree software. The tree file was associated with the trees depicted in figure 1 and supplementary figure S15.

**File Name:** Supplementary Data 5

**Description:** The Bayesian gene tree generated using MrBayes. The phylogenic tree was transformed as cladogram in NEXUS format and could be visualized using the FigureTree software. The tree file was associated with the trees depicted in figure 1 and supplementary figure S15.

**File Name:** Supplementary Data 6

**Description:** Synonymous substitutions per synonymous site (Ks) for duplicated paralog pairs detected on the *LEAFY* loci associated syntenic blocks in grape and rice genomes. The frequency distribution of synonymous divergence of syntelog pairs adjacent of the *LEAFY* loci were employed to evaluate the synonymous divergence level of the associated genomic syntenic blocks to trace the polyploidy events.

**File Name:** Supplementary Data 7

**Description:** The edge list and synteny scores of the synteny network for the LEAFY gene family. An edge in the network represented the syntenic relationship of the two LEAFY genes and thicker edges correlate with higher syntenic scores. All syntenic relationships among LEAFY loci were thus represented by a syntenic network. Gene accession information could be found in the supplementary data 1.
