# Supplementary material for: Ancient duplications and grass-specific transposition influenced the evolution of LEAFY transcription factor genes
Source: Commun Biol. 2019 Jun 21;2:237. doi: 10.1038/s42003-019-0469-4 (PMC6588583; doi:10.1038/s42003-019-0469-4)
Supplement: Supplementary file 10 — Reporting Summary [file 42003_2019_469_MOESM10_ESM.pdf]

nature research

Corresponding author(s): Jianhua Zhang

Last updated by author(s): Apr 27, 2019

Reporting Summary

Nature Research wishes to improve the reproducibility of the work that we publish. This form provides structure for consistency and transparency in reporting. For further information on Nature Research policies, see Authors & Referees and the Editorial Policy Checklist.

Statistics

For all statistical analyses, confirm that the following items are present in the figure legend, table legend, main text, or Methods section.

n/a	Confirmed	
☒	☐	The exact sample size (n) for each experimental group/condition, given as a discrete number and unit of measurement
☒	☐	A statement on whether measurements were taken from distinct samples or whether the same sample was measured repeatedly
☒	☐	The statistical test(s) used AND whether they are one- or two-sided<br>*Only common tests should be described solely by name; describe more complex techniques in the Methods section.*
☒	☐	A description of all covariates tested
☒	☐	A description of any assumptions or corrections, such as tests of normality and adjustment for multiple comparisons
☒	☐	A full description of the statistical parameters including central tendency (e.g. means) or other basic estimates (e.g. regression coefficient) AND variation (e.g. standard deviation) or associated estimates of uncertainty (e.g. confidence intervals)
☒	☐	For null hypothesis testing, the test statistic (e.g. F, t, r) with confidence intervals, effect sizes, degrees of freedom and P value noted<br>*Give P values as exact values whenever suitable.*
☐	☒	For Bayesian analysis, information on the choice of priors and Markov chain Monte Carlo settings
☒	☐	For hierarchical and complex designs, identification of the appropriate level for tests and full reporting of outcomes
☒	☐	Estimates of effect sizes (e.g. Cohen's d, Pearson's r), indicating how they were calculated

Our web collection on statistics for biologists contains articles on many of the points above.

Software and code

Policy information about availability of computer code

Data collection	Sequence data were primarily collected from Phytozome, oneKP and other relevant databases. Detailed information were described in methods section where appropriate.
Data analysis	HMMWER v3.1vb2 --- domain annotation and alignment;<br>Phyutility v2.2.6 --- sequence alignment trimming;<br>IQ-TREE v1.6.8 and Mrbayes v3.2.6 --- phylogenetic analyses;<br>Tracer v1.7.1 --- Posterior Summarization in Bayesian Phylogenetics;<br>FigTree v1.4.4 --- gene tree visualization;<br>MEGA v7.0.26 --- Ancestral State Reconstruction analyses;<br>Diamond v0.9.22.123 --- fast proteome comparisons;<br>MCScanX --- genomic synteny analyses;<br>Muscle v3.8.31 --- sequence alignment;<br>PAL2NAL v14 --- protein sequence alignment back-translation;<br>KaKs_calculator v2.0 --- Synonymous substitution rate estimation;<br>Cytoscape v3.7.0 --- Network visualization.

For manuscripts utilizing custom algorithms or software that are central to the research but not yet described in published literature, software must be made available to editors/reviewers. We strongly encourage code deposition in a community repository (e.g. GitHub). See the Nature Research guidelines for submitting code & software for further information.

Data

Policy information about availability of data

All manuscripts must include a data availability statement. This statement should provide the following information, where applicable:

- Accession codes, unique identifiers, or web links for publicly available datasets
- A list of figures that have associated raw data
- A description of any restrictions on data availability

All accessions of sequences included in this study were listed in the supplementary data. And all of them are available in pubic databases. We also provided the sequences and sequence alignments in the supplementary data.

Field-specific reporting

Please select the one below that is the best fit for your research. If you are not sure, read the appropriate sections before making your selection.

☐ Life sciences ☐ Behavioural & social sciences ☒ Ecological, evolutionary & environmental sciences

For a reference copy of the document with all sections, see nature.com/documents/nr-reporting-summary-flat.pdf

Ecological, evolutionary & environmental sciences study design

All studies must disclose on these points even when the disclosure is negative.

Study description	We used maximum-likelihood and Bayesian inference approaches to reconstruct the LEAFY gene family phylogeny, comprehensive inclusion of family member from mosses and liverworts unveiled a high-confidence gene duplication event before the functional divergency of type-I and type-II LEAFY genes in the ancestry of liverworts, mosses and tracheophytes, hornwort promiscuous genes are all outside of this duplication. Ancestral state reconstruction analyses suggested the ancestral state of LEAFY in embryophyte was promiscuous, supporting an subfunctionalization following duplication. The identification of promiscuous LEAFY from Osmunda (an early-diverging fern genus) suggested an ancient transient duplication in the ancestor of embryophytes. Comprehensive genome synteny comparisons among angiosperms demonstrated deep genomic positional conservation of LEAFY genes and uncovered an ancestral grass-specific transposition activity.
Research sample	To generate the broad-scale family phylogeny for LEAFY genes, family members in non-flowering plants were extensively mined from augmented OneKP transcriptomic data and incorporated with the angiosperm genes, members were identified from multiple streptophyte lineages including Charophytes (streptophytic algae), hornworts, liverworts, mosses, lycophytes, ferns, gymnosperms and angiosperms.
Sampling strategy	Gene family members were comprehensively collected from diverse plants lineages to cover all major clades of streptophytes.
Data collection	The target gene members were identified and collected according to experimentally determined functional domains.
Timing and spatial scale	Not applicable.
Data exclusions	No data was excluded.
Reproducibility	Accessions of the sequences are listed in a supplementary table. And all the bioinformatic analytical details (including software versions and parameters used) were described in the methods.
Randomization	For phylogenetic analyses, bootstrapping datasets were sampled at least 1000 times, and sufficient generations were sampled to ensure convergence of Bayesian MCMC runs were achieved, all of which represented the field standard.
Blinding	Not applicable.

Did the study involve field work? ☐ Yes ☒ No

Reporting for specific materials, systems and methods

We require information from authors about some types of materials, experimental systems and methods used in many studies. Here, indicate whether each material, system or method listed is relevant to your study. If you are not sure if a list item applies to your research, read the appropriate section before selecting a response.

Materials & experimental systems

n/a	Involved in the study
☒	☐ Antibodies
☒	☐ Eukaryotic cell lines
☒	☐ Palaeontology
☒	☐ Animals and other organisms
☒	☐ Human research participants
☒	☐ Clinical data

Methods

n/a	Involved in the study
☒	☐ ChIP-seq
☒	☐ Flow cytometry
☒	☐ MRI-based neuroimaging
